# Organic Synthesis via Transition Metal-Catalysis

**DOI:** 10.3390/molecules27041227

**Published:** 2022-02-11

**Authors:** Bartolo Gabriele

**Affiliations:** Laboratory of Industrial and Synthetic Organic Chemistry (LISOC), Department of Chemistry and Chemical Technologies, University of Calabria, Via Pietro Bucci 12/C, 87036 Arcavacata di Rende, Italy; bartolo.gabriele@unical.it

In recent years, the development of transition-metal-catalyzed reactions has acquired an increasing importance. In fact, the use of transition metal complexes as catalysts may allow performing important organic transformations in one synthetic step by the assembly of simple units under sustainable conditions [1]. This Special Issue reports significant contributions in this field. In particular, seven papers have been published: two reviews and five original research articles.

The first review (by Garazi Urgoitia, Maria Teresa Herrero, Fátima Churruca, Nerea Conde, and Raul SanMartin) reports on the use of palladium pincer complexes as catalysts or pre-catalysts for the direct arylation of arenes with aryl halides or pseudo-halides [2]. This is a particularly important process, which allows aryl–aryl coupling (also intramolecularly) using an arene (or heteroarene) as one coupling partner, with C–H activation.

The second review (by Mieko Arisawa and Masahiko Yamaguchi) concerns the synthesis of a plethora of organosulfur compounds by rhodium-catalyzed S–S bond cleavage of disulfides or elemental sulfur and subsequent transfer of the ensuing organothio groups to organic substrates [3].

In the first original research article, Jérémy Ternel, Adrien Lopes, Mathieu Sauthier, Clothilde Buffe, Vincent Wiatz, Hervé Bricout, Sébastien Tilloy, and Eric Monflier describe the reductive hydroformylation (performed under 80 bar pressure of a 1:1 CO/H_2_ mixture, in toluene at 80 °C) of isosorbide diallyl ether (readily available by allylation of bio-sourced isosorbide). The process is catalyzed by a rhodium/amine catalytic system, [typically, Rh(acac)(CO)_2_/Et_3_N], to give the corresponding high value-added bis-primary alcohols [4].

The second original research article (by Joanna Palion-Gazda, André Luz, Luis R. Raposo, Katarzyna Choroba, Jacek E. Nycz, Alina Bieńko, Agnieszka Lewińska, Karol Erfurt, Pedro V. Baptista, Barbara Machura, Alexandra R. Fernandes, Lidia S. Shul’pina, Nikolay S. Ikonnikov, and Georgiy B. Shul’pin) reports on the use of methyl-substituted 8-hydroxyquinolines (Hquin) for the preparation of five-coordinated oxovanadium(IV) complexes [VO(2,6-(Me)_2_-quin)_2_, VO(2,5-(Me)_2_-quin)_2_, and VO(2-Me-quin)_2_]. These complexes were then used as catalysts for the efficient oxidation of hydrocarbons (to alcohols) and alcohols (to ketones), carried out with H_2_O_2_ in acetonitrile at 50 °C in the presence of 2-pyrazinecarboxylic acid (PCA) as a cocatalyst [5].

The subsequent papers report on transition-metal-promoted cyclization processes leading to heterocyclic derivatives. Thus, new polycyclic heterocycles (1*H*-benzo[4,5]imidazo[1,2-*c*][1,3]oxazin-1-ones) were synthesized by Lucia Veltri, Roberta Amuso, Marzia Petrilli, Corrado Cuocci, Maria A. Chiacchio, Paola Vitale, and Bartolo Gabriele using a ZnCl_2_-promoted deprotective annulation approach starting from *N*-Boc-2-alkynylbenzimidazoles under mild conditions (CH_2_Cl_2_ as the solvent at 40 °C for 3 h) [6]. *N*-benzoylindoles were obtained by Zhe Chang, Tong Ma, Yu Zhang, Zheng Dong, Heng Zhao, and Depeng Zhao by Pd(II)-catalyzed oxidative C–H functionalization and annulation of substituted *N*-(2-allylphenyl)benzamides, carried out with Pd(OAc)_2_ as the catalyst in the presence of benzoquinone as the oxidant and dibutyl phosphate as the additive in DMSO at 60–70 °C [7]. An annulative C–H activation process was also developed by Bao Wang, Xu Han, Jian Li, Chunpu Li, and Hong Liu for the synthesis of fused isochromeno-1,2-benzothiazine derivatives, starting from *S*-phenylsulfoximides and 4-diazoisochroman-3-imine. The process, catalyzed by [Cp*RhCl_2_]_2_ in the presence of AgOPiv, takes place in trifluoroethanol at room temperature under air [8].
